# Friends, Take Notice, and Act

**Published:** 1872-03

**Authors:** 


### PART III.

### Editorials, Correspondence and Miscellaneous.

### FRIENDS, TAKE NOTICE, AND ACT!

Dr. J. S. Whorton, of Lynchburg, Virginia, in a letter under date of March 2nd, 1872, writes:

"I have had the volume for 1871 bound, and consider it invaluable as a book of reference, and well worth several times the price of subscription."

This is the general verdict of our subscribers of 1871. As stated long ago, a full set of volume No. 1 cannot now be furnished, yet the publisher has on hand six or eight numbers of that volume, which can be had by paying at the rate of the regular subscription price; and although incomple as a volume, will be of great benefit to the busy practitioner.

We intend to make the second volume *far superior* to the first, as a "Book of Reference," and one our friends should not neglect to secure; hence we earnestly desire them to commence with the first number of the volume. Our publisher has left on hand, of the very large edition of that number, *a few hundred copies only*, and we ask our subscribers to do their *personal friends* the favor to have them start with this number before it is exhausted.

